# Editorial: Managing chronic obstruction pulmonary disease: From translational research to public health practice

**DOI:** 10.3389/fmed.2022.965759

**Published:** 2022-08-19

**Authors:** Chia-Li Han, Kin-Fai Ho, Shu-Chuan Ho, Hsiao-Chi Chuang

**Affiliations:** ^1^Master Program in Clinical Genomics and Proteomics, College of Pharmacy, Taipei Medical University, Taipei City, Taiwan; ^2^JC School of Public Health and Primary Care, The Chinese University of Hong Kong, Shatin, Hong Kong SAR, China; ^3^School of Respiratory Therapy, College of Medicine, Taipei Medical University, Taipei City, Taiwan; ^4^Division of Pulmonary Medicine, Department of Internal Medicine, Shuang Ho Hospital, Taipei Medical University, Taipei City, Taiwan; ^5^Graduate Institute of Clinical Medicine, College of Medicine, Taipei Medical University, Taipei City, Taiwan; ^6^Cell Physiology and Molecular Image Research Center, Wan Fang Hospital, Taipei Medical University, Taipei City, Taiwan

**Keywords:** air pollution, emphysema, respiratory therapy, cell-based therapy, clinical management, imaging

Chronic obstructive pulmonary disease (COPD) is an important public health issue, which is the fourth leading cause of death in the world (1). Approximately 6% of all deaths (more than 3 million people) occurred as a result of COPD (2). Because of continuous exposure of COPD risk factors and aging of the population, the incidence of COPD is projected to increase in coming decades (3). Exposure to particles from cigarette smoke, occupational hazards, and air pollution are recognized as risk factors in the development and progression of COPD (2). It is worth noting that no effective treatment has been found that can fundamentally modify the disease and decrease the mortality of COPD currently, and health care of the disease often causes high medical costs.

Cigarette smoking is an important public health problem which has a direct effect on the respiratory system. Previous studies have demonstrated the harmful effect of smoking on the pulmonary function. Smoking accelerates decline in lung function, and often leads to COPD. Therefore, it is important to understand the possible ongoing impairment in lung function in smokers. In this special issue, Tian et al. reported that the annual decline rate of current male smokers with high smoking intensity (≥30 cigarettes per day) was 13.80 and 14.17 times greater than that of never-smokers in FEV1 and FVC. Moreover, a recent study indicated that lung function decline occurred in former smokers and low-intensity current smokers compared with never-smokers (4). All levels of smoking habit are probably linked with lung impairment and smoking cessation is the most effective way for risk reduction in COPD.

Emphysema, usually associated with cigarette smoking, is a phenotype of COPD in which alveoli become damaged and destroyed. But many people diagnosed with COPD have never smoked. Tung et al. investigated the relationship of various air pollutants with emphysema measured through high-resolution CT (HR-CT) lung scans and lung function testing. The results indicated that particulate matter <2.5 μm in aerodynamic diameter (PM_2.5_), nitrogen dioxide (NO_2_), and ozone (O_3_) were associated with an increased degree of upper lobe emphysema and lower lobe emphysema. It is important to explore factors that contribute to emphysema, particularly in a large, multi-ethnic group of adults. Moreover, the combined health effect of multiple air pollutants—PM_2.5_, NO_2_, and O_3_ can be addressed which can aid in our understanding and control of emphysema in COPD in the future.

Increasing reports showed the advantages of CT on quantification of COPD severity. Cao et al. identified that expiratory CT scans provided a more accurate assessment of COPD than the inspiratory CT scans. Also, the results of the quantitative parameter intrapulmonary vascular volume (IPVV) was significantly associated with FEV1%, emphysema degree and airway disease. Based on the powerful approaches with different advanced quantitative models, CT would provide more information regarding COPD severity for clinical diagnosis and treatment strategy.

Since the pathogenesis of COPD is unclear, there is no cure but pharmacological therapies to slow the progression of COPD. A multicenter prospective longitudinal study in China was conducted to evaluate the effectiveness of inhaled combination LABA/LAMA treatment and triple (ICS/LABA/LAMA) therapy in a total of 695 symptomatic COPD patients *via* assessing the minimum clinical important difference (MCID) defined by attaining a COPD assessment test decrease ≥2 (Cheng et al.). Nearly 50% of patients attained MCID, especially the female patients. Among these, patients treated with LABA/LAMA or ICS/LABA/LAMA were more likely to attain MCID than patients treated with LAMA monotherapy. A higher incidence of severe exacerbations was observed in patients treated with LABA/LAMA than those with ICS/LABA/LAMA. Apart from the approved inhalation therapy, Chen et al. reported the usage of human umbilical cord-derived mesenchymal stem cells (MSCs) in treating the mouse model of cigarette smoke-induced COPD emphysema. A number of inflammatory molecules were found to be decreased not only locally in the lung tissues but also systematically in serum after MSC administration. Significant reduction in emphysema severity was also observed, suggesting the immunoregulation and repair potential of MSCs in treating COPD. In addition, a novel long non-coding RNA, Nqo1 antisense transcript 1 (Nqo1-AS1), was reported by Zhang et al. to attenuate the cigarette smoke-induced oxidative stress by increasing the Serpina mRNA expression as well as the protein level of Nqo1 through stabilizing its mRNA.

COPD is a chronic inflammatory disease of the lung associated with the structural remodeling of airways and irreversible airflow obstruction caused by various factors. With the advanced CT with quantification models, precision medicine for diagnosis of COPD with emphysema could be conducted for evaluating disease stability and severity. In addition to traditional strategies for COPD management, an increasing development of molecular drugs and stem cell therapy provides a bright future for patient welfare and quality of life. Taken together, this Research Topic has pushed forward our understanding of COPD in terms of risk factors, pathophysiology, diagnosis, and treatment.

## Author contributions

C-LH, K-FH, S-CH, and H-CC drafted and revised this editorial. All authors contributed to the article and approved the submitted version.

## Conflict of interest

The authors declare that the research was conducted in the absence of any commercial or financial relationships that could be construed as a potential conflict of interest.

## Publisher's note

All claims expressed in this article are solely those of the authors and do not necessarily represent those of their affiliated organizations, or those of the publisher, the editors and the reviewers. Any product that may be evaluated in this article, or claim that may be made by its manufacturer, is not guaranteed or endorsed by the publisher.

## References

[1] Vestbo J Hurd SS Agustí AG Jones PW Vogelmeier C Anzueto A. Global strategy for the diagnosis, management, and prevention of chronic obstructive pulmonary disease. Am J Respir Crit Care Med. (2013) 187:347–65. 10.1164/rccm.201204-0596PP 22878278

[2] Global Initiative for Chronic Obstructive Lung Disease. Global Strategy for Prevention, Diagnosis and Management of COPD (2020 report). (2020). GOLD

[3] Mathers CD Loncar D. Projections of global mortality and burden of disease from 2002 to 2030. PLoS Med. (2006) 3:e442. 10.1371/journal.pmed.0030442 17132052 PMC1664601

[4] Oelsner EC Balte PP Bhatt SP Cassano PA Couper D Folsom AR. Lung function decline in former smokers and low-intensity current smokers: a secondary data analysis of the NHLBI Pooled Cohorts Study. Lancet Respir Med. (2020) 8:34–44. 10.1016/S2213-2600(19)30276-0 31606435 PMC7261004

